# Effect of Calcifediol on Physical Performance and Muscle Strength Parameters: A Systematic Review and Meta-Analysis

**DOI:** 10.3390/nu14091860

**Published:** 2022-04-29

**Authors:** Mario Barbagallo, Nicola Veronese, Agnese Di Prazza, Francesco Pollicino, Luca Carruba, Anna La Carrubba, Ligia J. Dominguez

**Affiliations:** 1 Geriatric Unit, Department of Internal Medicine and Geriatrics, University of Palermo, 90127 Palermo, Italy; mario.barbagallo@unipa.it (M.B.); nicola.veronese@unipa.it (N.V.); agnesediprazza@gmail.com (A.D.P.); francesco_pollicino@yahoo.it (F.P.); luca.carruba1992@gmail.com (L.C.); lacarrubbaanna@gmail.com (A.L.C.); 2 Faculty of Medicine and Surgery, Kore University of Enna, 94100 Enna, Italy

**Keywords:** vitamin D, calcifediol, sarcopenia, physical performance, muscle strength, meta-analysis

## Abstract

There is general agreement that optimal vitamin D status is necessary for bones, muscles, and general health, particularly in older adults, who are at higher risk of negative consequences of vitamin D deficiency, including sarcopenia; vitamin D supplementation is proposed as a potential intervention to mitigate sarcopenia. Several RCTs have reported that calcifediol (25(OH)D) was more potent than cholecalciferol in increasing plasma 25(OH)D. The present systematic review and meta-analysis aimed to summarize the effects of calcifediol on physical performance and muscle strength. We searched databases from inception to 1 January 2022 for studies investigating calcifediol on physical performance or muscle strength parameters. We calculated the difference between the means of follow-up vs. baseline data using standardized mean differences (SMD) and their 95% confidence intervals (CIs); a random-effect model was considered for all of the analyses. Seven RCTs were included in the meta-analysis. Calcifediol significantly improved gait speed (SMD = 2.500; 95%CI = 1.768–3.223; *p* < 0.0001); handgrip strength (*n* = 5446 participants, SMD = 0.532; 95%CI: 0.305–0.758; *p* < 0.0001; I^2^ = 20.2%); and leg extension (*n* = 4318 participants, SMD = 0.641; 95%CI: 0.346 to 0.935; *p* < 0.0001; I^2^ = 18.8%;) vs. baseline values. In conclusion, in this systematic review and meta-analysis, we observed that calcifediol may have a positive effect on muscle strength parameters, with less evidence on physical performance. These data further indicate the importance of vitamin D and, in particular, of calcifediol, not only on bone metabolism but also on muscle parameters and sarcopenia.

## 1. Introduction

The interest in vitamin D has increased in the last years, since growing literature has reported that the deficit of vitamin D (i.e., hypovitaminosis D) could be associated with several negative health outcomes [1]. Moreover, hypovitaminosis D is a highly frequent condition, particularly in older populations [2].

Whilst the evidence regarding the effects of the vitamin D hormone on bone at all ages is solid, the possible extra-skeletal benefits of vitamin D supplementation [1], for example, on muscle, are still debated.

The potential association between hypovitaminosis D and sarcopenia, i.e., the loss of muscle mass, poor physical performance, and low muscle strength, has been reported by several epidemiological studies [3,4,5]. However, whether the supplementation with vitamin D metabolites can prevent physical performance and muscle strength reduction is still unclear [6]. In a former systematic review regarding vitamin D supplementation and physical performance and muscle strength parameters, the authors found conflicting results: for example, they found four intervention trials reporting a significant effect of vitamin D on muscle strength, but the effect was not observed in the other three studies [3]. It is worth mentioning that the preparations available for vitamin D supplementation, i.e., vitamin D of plant origin (D2, ergocalciferol), that of animal origin (D3, cholecalciferol), and calcifediol (25 hydroxyvitamin D, 25(OH)D, also known as calcidiol) have different pharmacokinetic characteristics and potency [2]. One possible limitation of this previous research [3] is that almost all of the studies included in the systematic review used cholecalciferol or ergocalciferol as vitamin D supplementation; only one used the active form calcitriol (1,25(OH)_2_D), and none used calcifediol.

On the contrary, several randomized controlled trials (RCTs) on vitamin D supplementation have found that calcifediol was more potent in the correction of poor vitamin D status, and this correction was achieved in a shorter time compared to cholecalciferol [2]. Indeed, from a pharmacokinetic point of view, calcifediol is more potent than cholecalciferol [2,7]. However, the potential effect of calcifediol on physical performance and muscle strength parameters is poorly known, even if sarcopenia is particularly frequent and relevant in geriatric medicine [8], being associated with several negative outcomes [9].

Given this background, the present systematic review and meta-analysis aimed to summarize the current state of the art of the effects of calcifediol on physical performance and muscle strength parameters.

## 2. Materials and Methods

### 2.1. Registration Statement

This systematic review adhered to the PRISMA statement [10] and followed a pre-planned protocol available at https://osf.io/9km7y/ (accessed on 20 April 2022) and in the Supplementary Material.

### 2.2. Data Sources and Searches

Two investigators (NV and LJD) conducted a literature search using PubMed/Medline and Scopus from database inception until 1 January 2022.

In PubMed, the following search strategy was used: “(Calcifediol OR Calcidiol OR Dedrogyl OR Didrogyl) AND (physical performance OR gait speed OR walking speed OR handgrip strength OR strength OR sarcopenia OR frailty OR muscle strength)”. Any inconsistencies were resolved by consensus with a third senior author (MB).

### 2.3. Study Selection

We used the following PICOS question, as reported in the Supplementary Material: in people with hypovitaminosis D (P), compared to baseline values (C), what is the effect of calcifediol (I) on muscle strength and physical performance parameters (O), based on evidence derived from intervention and observational studies (S)?

Inclusion criteria for this meta-analysis were: (i) use of oral calcifediol; (ii) reporting information on physical performance (i.e., tests more depending on aerobic capacity than muscle power) [11] or muscle strength (i.e., tests more depending on muscle power than aerobic capacity) [11] outcomes; (iii) written in English. Studies were excluded if: (i) did not include humans; (ii) lack of sufficient information for performing a meta-analysis. In this later case, the corresponding/first author of the article was contacted through e-mail. Study selection, at title/abstract and full-texts level, was made using Rayyan (https://www.rayyan.ai/, accessed on 1 January 2022).

### 2.4. Data Extraction

Five independent investigators (NV, ADP, FP, LC, ALC) extracted key data from the included articles in a standardized Excel spreadsheet with two senior authors (LJD and MB) checking the data. For each article, we extracted data on author names, year of publication, country, condition, daily calcifediol supplementation (reported in microgram, µg), demographic information (mean age, females (%)), mean serum 25(OH)D levels reported for all studies in nmol/L (if originally reported in ng/mL, please multiply by 2.496) at baseline and follow-up, follow-up in weeks.

### 2.5. Outcomes

The primary outcomes considered were the changes (between follow-up and baseline) in physical performance tests. The included tests for physical performance were chair rise time, gait/walking speed, short physical performance battery (SPPB), and timed up and go. The tests of muscle strength included were leg extension, leg flexion, and handgrip strength. Finally, the changes between follow-up and baseline values of 25(OH)D levels were considered as outcomes.

### 2.6. Quality Assessment

For assessing the quality of the studies included, since studies reporting a pre-post comparison without a control group were analyzed, we used a specific tool suggested by the National Heart, Lung, Blood Institute (NHLBI) based on twelve different questions and available at https://www.nhlbi.nih.gov/health-topics/study-quality-assessment-tools (accessed on 1 January 2022).

### 2.7. Data Synthesis and Analysis

All analyses were performed using STATA version 14.0 (StataCorp, College Station, TX, USA).

The primary analysis compared the changes in physical performance and muscle strength parameters between follow-up vs. baseline values. We then calculated the difference between the means of follow-up vs. baseline data using standardized mean differences (SMD) and their 95% confidence intervals (CIs). A random-effect model was considered for all analyses, being the less conservative and hypothesizing several clinical differences across the studies included [12].

Heterogeneity across works was assessed by the I^2^ metric and χ^2^ statistics. Given significant heterogeneity (I^2^ ≥ 50%, *p* < 0.05), a series of meta-regression analyses was planned, according to follow-up (weeks), mean age, percentage of females, serum 25(OH)D levels (baseline, follow-up, changes), and dosages of calcifediol (10, 20, 30 µg/daily).

Publication bias was assessed by visually inspecting funnel plots and using the Begg–Mazumdar Kendall tau [13] and the Egger bias test [14].

For all analyses, a *p*-value less than 0.05 was considered statistically significant.

## 3. Results

### 3.1. Search Results

**Figure 1:**
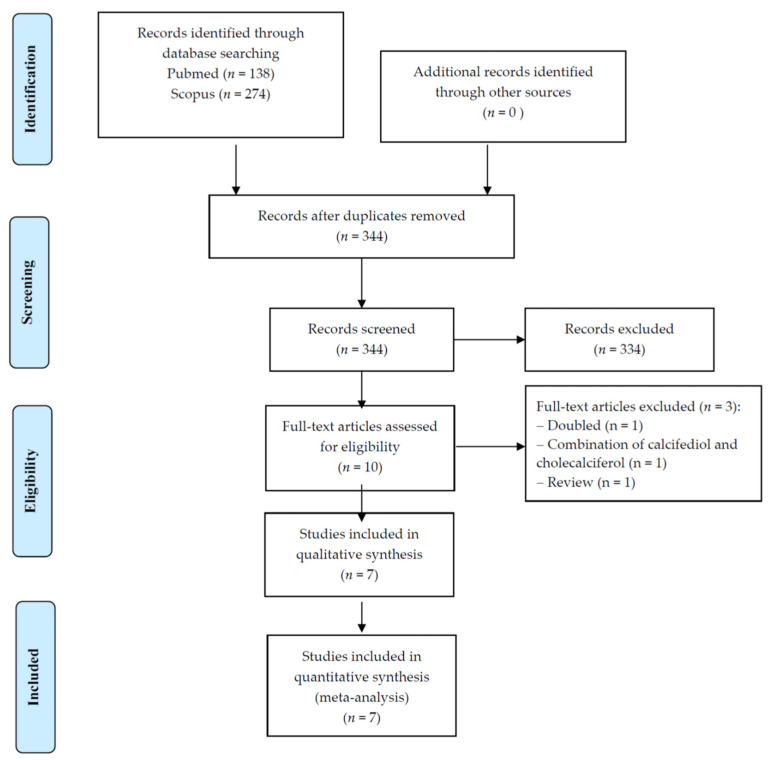
PRISMA flow-chart.

As shown in Figure 1, among 344 records initially screened, ten were retrieved as full-texts: of them, seven works were included in the systematic review and meta-analysis [15,16,17,18,19,20,21].

### 3.2. Study and Participants Characteristics

Overall, the seven works followed up 269 participants for a median of 24 weeks. The mean age was 67.4 ± 7.1 years, and the participants were mainly females. The studies were mostly conducted in Italy (*n* = 4) and the Netherlands (*n* = 2), whilst only one was conducted in the USA. Among the seven studies included, five studies used calcifediol at a dosage of 20 µg/daily, one of 30 µg, and the other two of 10 µg, in similar dosage form. Considering all of the studies, we observed a significant increase in the mean serum levels of 25(OH)D from 39 ± 11.4 at baseline to 132 ± 30 nmol/L at the follow-up.

The main characteristics of the studies included are depicted in Table 1.

### 3.3. Meta-Analysis of Calcifediol on Physical Performance and Muscle Strength Parameters

Table 2 shows the effect of calcifediol on physical performance and muscle strength parameters.

**Figure 2:**
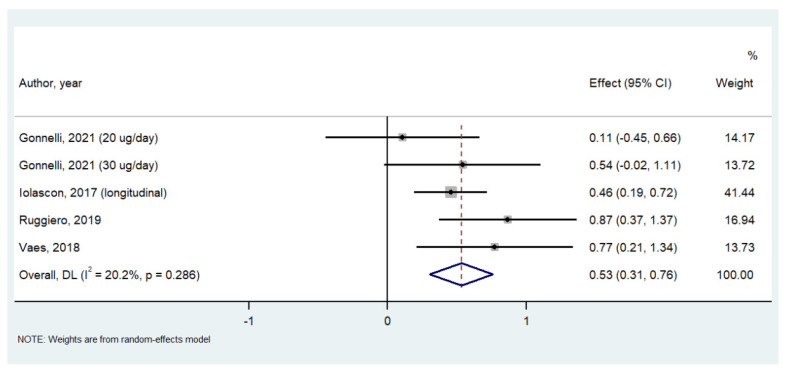
Forrest plot of the effect of calcifediol on handgrip strength. References [16,18,19,21].

**Figure 3:**
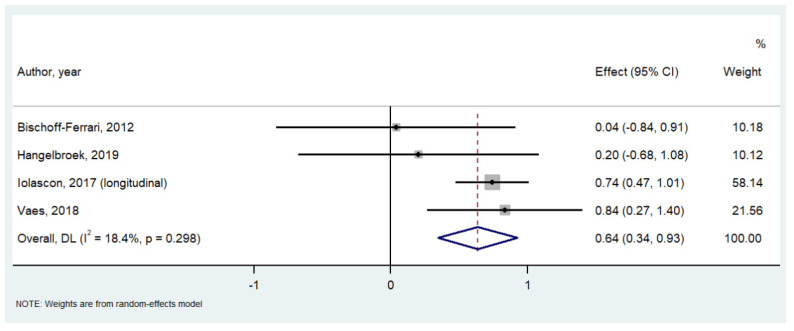
Forrest plot of the effect of calcifediol on leg extension. References [15,17,18,21].

Calcifediol significantly improved gait speed in one study including 52 participants (SMD = 2.500; 95%CI = 1.768–3.223; *p* < 0.0001), whilst no effect was observed for the other physical performance tests included. On the contrary, calcifediol significantly improved handgrip strength (*n* = 5446 participants, SMD = 0.532; 95%CI: 0.305–0.758; *p* < 0.0001; I^2^ = 20.2%; Figure 2) and leg extension (*n* = 4318 participants, SMD = 0.641; 95%CI: 0.346 to 0.935; *p* < 0.0001; I^2^ = 18.8%; Figure 3) compared to the baseline values.

No publication bias emerged for any test included.

### 3.4. Meta-Regression Analysis

Since no outcome having at least four studies—the minimum number of studies for running a meta-regression analysis—had a high heterogeneity (I^2^ = 50%), meta-regression analyses were not made.

### 3.5. Adverse Events

Adverse events were reported in some of the studies included. In the study of Bischoff-Ferrari et al. [17], in the group treated with calcifediol, no cases of hypercalcemia were observed, even if serum calcium was assessed several times during the follow-up. Similarly, Iolascon et al. [18] did not report any side effects during six months of follow-up. Finally, Vaes et al. [21] reported one or more mild adverse events in 39% of the patients treated with calcifediol; however, no differences were reported compared to placebo.

### 3.6. Risk of Bias Assessment

As shown in Supplementary Table S1, the studies included suffer on some potential biases, including the lack of a priori power assessment in almost all of the studies, the lack of information in comparing the interventions before and after calcifediol administration, and that the outcomes of interest were often taken only at baseline and at follow-up, without intermediate evaluations.

## 4. Discussion

In this systematic review and meta-analysis of the literature regarding effects of calcifediol on physical performance and muscle strength parameters, we found that, over a median administration of 24 weeks, calcifediol significantly improved several muscle strength parameters and some physical performance tests. Of importance, the studies were not statistically heterogeneous and not affected by any publication bias. These findings suggest that calcifediol, also independently from the dose given, is able to improve muscle performance, potentially suggesting its use in the prevention and treatment of sarcopenia, a relevant condition in older adults.

The term vitamin D is generic, as it refers to a group of fat-soluble compounds with a main chain of cholesterol rings including vitamin D2 (ergocalciferol, of plant origin) and vitamin D3 (cholecalciferol, of animal origin). Once vitamin D (D2 and D3) reaches the circulation, it is metabolized into 25(OH)D (calcifediol), mainly in the liver, thanks to the action of various hydroxylases, but this can occur in a variety of tissues in autocrine/paracrine modality [22]. Afterwards, hydroxylation occurs in the renal tubule to produce the active molecule 1,25(OH)_2_D (calcitriol), which interacts with vitamin D receptor (VDR), or the inactive metabolite 24,25(OH)_2_D [22]. Even if calcitriol is the active form of the vitamin/hormone D, its use is indicated only in patients with decreased calcitriol synthesis due to chronic renal failure or in genetic disease (i.e., type 1 vitamin D-dependent rickets) [23,24,25]. Calcitriol is associated with a high incidence of hypercalcemia, and it is not recommended for general vitamin D supplementation [2].

It is noteworthy that the rate of conversion of vitamin D (D2 and D3) to 25(OH)D (calcifediol) may be slower in people receiving high doses of vitamin D (D2 and D3) [26], which may help to explain why high loading doses of vitamin D (D2 and D3) are not currently recommended and have been associated with significant adverse effects [27,28,29].

The available preparations for vitamin D supplementation, i.e., ergocalciferol (D2), cholecalciferol (D3), and calcifediol (25(OH)D) have different pharmacokinetic characteristics and potency. Cholecalciferol has a half-life of about two days, calcifediol of three weeks, and calcitriol of a few hours [26]. Calcifediol has a faster absorption, which can be explained, because it occurs through the portal vein circulation as opposed to the more complex lymphatic pathway used by cholecalciferol. This transportation difference may (at least in part) explain the greater bioavailability [30]. As opposed to cholecalciferol, calcifediol has been reported to have a linear absorption when administered in daily or weekly schedules. In a study of postmenopausal women receiving calcifediol for three months, 25(OH)D serum levels were raised without modifications in other parameters of mineral metabolism, and the magnitude of absolute percentage increase was similar for those with baseline levels below or above 20 ng/mL [31].

The chief determinant of the length of time that a vitamin D metabolite remains in the circulation is its affinity to vitamin D binding protein (DBP) [32]. The dissociation constant of this binding, which is different in calcifediol and cholecalciferol [2], determines the free concentrations, which allows the molecule’s diffusion across the cell membrane and, thereby, the cellular activity. Thus, metabolite–DBP binding sustains stable levels of vitamin D metabolites and regulates their bioavailability, activation, and reactivity of the target organs [22].

Supplementation with calcifediol has been reported to correct poor vitamin D status in multiple studies in an efficient manner [2]. Several RCTs with different designs (six double-blind RCTs and seven open-label RCTs) have compared the ability of calcifediol with that of cholecalciferol to increase serum 25(OH)D concentrations. Even if the studies used different dosages (single or multiple), were conducted in heterogeneous populations, and in general included not a very high number of participants, all reported that calcifediol was more potent than cholecalciferol (two- to eight-fold) and that its use resulted in a faster increase of serum 25(OH)D [2]. A recent phase III-IV, double-blind, multicenter RCT assessed the efficacy and safety of calcifediol 0.266 mg soft capsules in vitamin D-deficient postmenopausal women (25(OH)D < 20 ng/mL) compared to cholecalciferol. Patients with baseline levels of serum were randomized 1:1:1 to calcifediol 0.266 mg/month for 12 months, calcifediol 0.266 mg/month for four months followed by placebo for eight months, and cholecalciferol 25,000 IU/month for 12 months. At month 4, 35% of postmenopausal women treated with calcifediol and 8.2% of those treated with cholecalciferol reached serum 25(OH)D levels above 30 ng/mL (*p* < 0.0001). No relevant treatment-related safety issues were reported in any of the groups studied [33]. All these results confirm that calcifediol is effective, faster, and more potent than cholecalciferol in raising serum 25(OH)D levels and is a valuable option for the treatment of vitamin D deficiency.

One pharmacokinetic study suggested that it takes approximately 68 days with 800 IU/day of cholecalciferol to achieve the optimal plateau level [34]. This time could be reduced by increasing the dose or using a high bolus-loading dose, with the purpose of reaching the recommended levels of serum 25(OH)D for skeletal and general health in a relatively short period of time [35]. However, even if high doses of cholecalciferol seem to be safe regarding hypercalciuria and hypercalcemia [36], the most recent guidelines recommend not to use them due to possible adverse effects [37]. In fact, some studies have also shown that high-bolus doses ≥ 100,000 IU of cholecalciferol significantly increased bone resorption markers in a dose-dependent manner [37,38].

From a pathophysiological point of view, research in animal models has reported that treatment with vitamin D may increase the functional restoration of the injured muscle, decrease cellular apoptosis, and increase cellular proliferation [39]. Moreover, in vitro research has reported that the expression of the vitamin D receptor (VDR) linearly decreases with age [40], probably attenuating the effect of vitamin D metabolites on muscle physiology. Therefore, in order to have a positive effect on muscle, the circulating concentrations of vitamin D should be adequate and probably higher than in younger people. Our study clearly showed that in about six months of follow-up after calcifediol treatment, the mean increase in plasma 25(OH)D was about 100 nmol/L, suggesting that calcifediol is highly effective in raising this parameter, which is probably necessary for having a positive effect on muscle metabolism [41]. This significantly more potent and faster increase in plasma 25(OH)D has been repeatedly demonstrated in various populations [2].

Regarding the possible mechanisms by which vitamin D is related to muscle function, there is evidence in experimental studies indicating that vitamin D deficiency is associated with lower VDR content, increased oxidative stress, and altered activity of antioxidant enzymes in skeletal muscle [42]. Furthermore, it has been reported that vitamin D deficiency may induce paraspinal muscle atrophy and reduction in intramyonuclear VDR concentrations and VDR gene expression [43], while vitamin D plays a key role in the regulation of mitochondrial oxygen consumption and dynamics [44]. Thus, deficiency of vitamin D seems to decrease oxygen consumption rate and induce disruption of mitochondrial function, which renders it likely that vitamin D deficiency in the long run induces VDR ablation, ROS generation, and consequent deleterious effects on the mitochondrial function, possibly triggering muscle atrophy.

Among a number of factors that can increase the risk of sarcopenia in older adults, vitamin D deficiency is a key one [5]. Furthermore, it is easy to reverse by means of supplementation, even if several factors can limit the assumption and the metabolism of this important hormone, including less sun exposure and difficulties in eating foods containing vitamin D [21]; therefore, supplementation must be considered in many older people for both the prevention and treatment of sarcopenia [2,21], especially with molecules proven to be more potent in their ability to raise 25(OH)D levels with lower doses [2].

There is general agreement on the need for reaching and maintaining adequate levels of vitamin D for bones and general health among various scientific societies and international agencies. Nevertheless, the appropriate clinical ranges and method for restoring optimal levels are not yet univocal. For example, some indicate recommended circulating 25(OH)D levels to be over 20 ng/mL, while others consider adequate levels as over 30 ng/mL. However, all agree that a level below 10 ng/mL should be considered severe deficit [2]. A study examining 675 iliac crest biopsies from male and female patients did not find pathologic accumulation of osteoid in any patient with circulating 25(OH)D above 30 ng/mL [45]; thus, it is reasonable to suggest that the prescribed dose of vitamin D supplementation should ensure achieving this level of 25(OH)D in order to maintain skeletal health. In addition, treatments are based on the clinician experience and the availability of diverse vitamin D preparations.

A recent systematic review and meta-analysis of ten RCTs compared the effect of vitamin D supplementation (as monotherapy) with placebo on indices of sarcopenia in older (>50 years) adults. The authors reported that vitamin D supplementation conferred no effect on diverse indices of sarcopenia, including handgrip strength, short physical performance battery, timed up and go, and appendicular lean mass [46]. However, as opposed to our meta-analysis, all of the studies included used cholecalciferol, and none used calcifediol. This may reinforce the likelihood that a more potent molecule which makes it possible to reach higher circulating concentrations of 25(OH)D in a shorter period of time may be more effective in influencing muscle strength indices. Nevertheless, further studies are warranted with different molecules in order to make definitive conclusions.

Our systematic review shows that the effect of calcifediol is more evident for muscle strength parameters than for physical performance. We can justify these findings from a methodological point of view, since fewer studies regarding physical performance tests were available compared to muscle strength parameters, but our data agree with another classical meta-analysis showing the effects of vitamin D supplementation on muscle strength [47]. From a pathophysiological point of view, we can argue that VDRs are more present in type II muscular fibers (i.e., fibers that are important in muscle strength parameters) than in type I (i.e., long duration contractile fibers found in abundance in elite endurance athletes) [48,49].

Our systematic review has some strengths, such as being the first comprehensive work to systematically investigate the effect of calcifediol on two important aspects of older people, i.e., muscle strength and physical performance parameters. The results of the present meta-analysis should be considered taking into account its limitations. First, the RCTs included were small in sample size and used different dosages of calcifediol. Second, we were able to compare only pre/post physical performance and muscle strength parameters and not versus other interventions (such as cholecalciferol) or placebo, due to a limited number of studies available. Moreover, the genetic susceptibility to calcifediol was not explored, even if some recent works suggested that it is an important determinant of the final effect of vitamin D metabolites on muscle and bone [50]. Finally, the studies included had a limited follow-up time. Therefore, other larger studies with a longer follow-up are needed to confirm our observations.

## 5. Conclusions

This systematic review and meta-analysis reported that calcifediol may have a positive effect on muscle strength parameters and, with lesser evidence, on physical performance. Overall, our study further encourages the use of calcifediol in people with muscle strength weakness for improving this important aspect, often compromised in older people, and indicates the importance of vitamin D and, in particular, of calcifediol, not only on bone metabolism but also in sarcopenia.

## Figures and Tables

**Table 1:** Descriptive findings of the studies included.

Author, Year	Country	Sample Size	Condition	Daily Calcifediol Supplementation	Mean Age (SD)	Females (%)	Mean Serum 25(OH)D Levels (SD) nmol/L (Baseline vs. Follow-Up)	Follow-Up (Weeks)
Bischoff-Ferrari, 2012 [17]	USA	10	Healthy	20 µg	59.5 (6.3)	100	34.1 (9.1) 173.68 (11.9)	16
Corrado, 2021 [20]	Italy	26	Post-menopausal	20 µg	60.9 (8.1)	100	37.93 (11.8) 167 (108.0)	24
Gonnelli, 2021 (20 µg/day) [16]	Italy	25	Osteopenia/ osteoporosis	20 µg	62.4 (7.4)	100	40.5 (12.8) 148.3 (12.5)	24
Gonnelli, 2021 (30 µg/day) [16]	Italy	25	Osteopenia/ osteoporosis	30 µg	61.5 (8.3)	100	30.7 (10.2) 180.8 (12.5)	24
Hangelbroek, 2019 [15]	The Netherlands	10	Frailty	10 µg	71.8 (5.7)	40	69.9 (18.3) 87.3 (20.6)	24
Iolascon, 2017 (longitudinal) [18]	Italy	113	Osteoporosis	20 µg	68.0 (9.1)	100	27.1 (18.3) 105.5 (27.6)	24
Ruggiero, 2019 [19]	Italy	34	Hospitalized for any cause	20 µg	82.1 (5.7)	22	33.3 (1.3) 91.3 (42.3)	32
Vaes, 2018 [21]	The Netherlands	26	Frailty/ pre-frailty	10 µg	73.1 (6.0)	46	38.1 (2.9) 100 (5)	24
**Total**		**269**	**Osteopenia/osteoporosis, *n* = 3; frailty/pre-frailty, *n* = 2; hospital, *n* = 1; post-menopausal, *n* = 1; healthy, *n* = 1**	**30 µg, *n* = 1; 20 µg, *n* = 5; 10 µg, *n* = 2**	**67.4** **(7.1)**	**Only females. *n* = 5; mixed, *n* = 3**	**39 (11.4)** **132 (30)**	**Median = 24**

25(OH)D: hydroxyvitamin D; SD: standard deviation; USA: United States of America.

**Table 2:** Effect of calcifediol oral supplementation on physical performance and muscle strength parameters.

Parameter	Number of Comparisons	Number of Participants	SMD	95% CI		*p* Value	I^2^	Egger’s Test (*p*-Value)
**Chair rise time**	2	72	0.759	−0.980	2.499	0.39	90.4	Not possible
**Gait speed**	1	52	2.500	1.768	3.232	<0.0001	−	Not possible
**SPPB**	2	278	−0.012	−1.237	1.213	0.99	93.6	Not possible
**Timed up and go**	3	124	−0.264	−3.412	2.883	0.87	97.9	−35.8 (*p* = 0.63)
**Handgrip strength**	5	446	0.532	0.305	0.758	<0.0001	20.2	0.75 (*p* = 0.63)
**Leg extension**	4	318	0.641	0.346	0.935	<0.0001	18.8	0.75 (*p* = 0.69)
**Leg flexion**	3	92	0.304	−0.791	1.399	0.59	82.9	4.02 (*p* = 0.20)

CI: confidence interval; SMD: standardized mean differences; SPPB: short physical performance battery.

## Data Availability

The data and the databases are available upon reasonable request to the corresponding author.

## Supplementary Materials

The following supporting information can be downloaded at: https://www.mdpi.com/article/10.3390/nu14091860/s1, Table S1: Quality assessment of the studies included; Protocol: Effect of Calcifediol on Physical Performance and Muscle Strength Parameters: a Systematic Review and Meta-Analysis.
