# Supplementary material for: Effect of Calcifediol on Physical Performance and Muscle Strength Parameters: A Systematic Review and Meta-Analysis
Source: Nutrients. 2022 Apr 29;14(9):1860. doi: 10.3390/nu14091860 (PMC9103966; doi:10.3390/nu14091860)
Supplement: Supplementary file 1 [file nutrients-14-01860-s001.zip › nutrients-1659029-supplementary.pdf]

nutrients

MDPI

Article

Effect of Calcifediol on Physical Performance and Muscle Strength Parameters: A Systematic Review and Meta-Analysis

Mario Barbagallo [1], Nicola Veronese [1], Agnese Di Prazza [1], Francesco Pollicino [1], Luca Carruba [1], Anna La Carrubba [1] and Ligia J. Dominguez [1,2,*]

1 Geriatric Unit, Department of Internal Medicine and Geriatrics, University of Palermo, 90127 Palermo, Italy; mario.barbagallo@unipa.it (M.B.); nicola.veronese@unipa.it (N.V.); agnesediprazza@gmail.com (A.D.P.); francesco_pollicino@yahoo.it (F.P.); luca.carruba1992@gmail.com (L.C.); lacarrubbaanna@gmail.com (A.L.C.)

2 Faculty of Medicine and Surgery, Kore University of Enna, 94100 Enna, Italy

* Correspondence: ligia.dominguez@unipa.it or ligia.dominguez@unikore.it

Citation: Barbagallo, M.; Veronese, N.; Di Prazza, A.; Pollicino, F.; Carruba, L.; La Carruba, A.; Dominguez, L.J. Effect of Calcifediol on Physical Performance and Muscle Strength Parameters: A Systematic Review and Meta-Analysis. *Nutrients* **2022**, *14*, 1860. https://doi.org/10.3390/nu14091860

Academic Editors: José Manuel Quesada-Gómez and Roger Bouillon

Received: 14 March 2022

Accepted: 25 April 2022

Published: 29 April 2022

Publisher's Note: MDPI stays neutral with regard to jurisdictional claims in published maps and institutional affiliations.

Copyright: © 2021 by the authors. Submitted for possible open access publication under the terms and conditions of the Creative Commons Attribution (CC BY) license (https://creativecommons.org/licenses/by/4.0/).

Supplementary Table S1. Quality assessment of the studies included

Question	Bischoff-Ferrari, 2012	Corrado, 2021	Gonnelli, 2021	Hangelbroek, 2019	Iolascon, 2017	Ruggiero, 2019	Vaes, 2018
1. Was the study question or objective clearly stated?	Yes	Yes	Yes	Yes	Yes	Yes	Yes
2. Were eligibility/selection criteria for the study population prespecified and clearly described?	Yes	Yes	Yes	Yes	Yes	Yes	Yes
3. Were the participants in the study representative of those who would be eligible for the test/service/intervention in the general or clinical population of interest?	Yes	Yes	Yes	Yes	Yes	Yes	Yes
4. Were all eligible participants that met the prespecified entry criteria enrolled?	Yes	Yes	Yes	Yes	Yes	Yes	Yes
5. Was the sample size sufficiently large to provide confidence in the findings?	No	No	No	No	No	No	Yes
6. Was the test/service/intervention clearly described and delivered consistently across the study population?	Yes	Yes	Yes	Yes	Yes	Yes	Yes
7. Were the outcome measures prespecified, clearly defined, valid, reliable, and assessed consistently across all study participants?	Yes	Yes	Yes	Yes	Yes	Yes	Yes
8. Were the people	Yes	Yes	Yes	Yes	No	Yes	Yes

Question	Bischoff-Ferrari, 2012	Corrado, 2021	Gonnelli, 2021	Hangelbroek, 2019	Iolascon, 2017	Ruggiero, 2019	Vaes, 2018
**assessing the outcomes blinded to the participants' exposures/interventions?**							
**9. Was the loss to follow-up after baseline 20% or less? Were those lost to follow-up accounted for in the analysis?**	Not available	Yes	Yes	Not available	Yes	Not available	Yes
**10. Did the statistical methods examine changes in outcome measures from before to after the intervention? Were statistical tests done that provided p values for the pre-to-post changes?**	No	No	No	No	Yes	No	No
**11. Were outcome measures of interest taken multiple times before the intervention and multiple times after the intervention (i.e., did they use an interrupted time-series design)?**	No	No	Yes	No	No	No	Yes
**12. If the intervention was conducted at a group level (e.g., a whole hospital, a community, etc.) did the statistical analysis take into account the use of individual-level data to determine effects at the group level?**	NA	NA	NA	NA	NA	NA	NA

Supplementary Material

Protocol: Effect of Calcifediol on Physical Performance and Muscle Strength Parameters: a Systematic Review and Meta-Analysis

Authors:
Mario Barbagallo [1], Nicola Veronese [1], Agnese Di Prazza [1], Francesco Pollicino [1], Luca Carruba [1], Anna La Carruba [1] and Ligia J. Dominguez [1,2,*]

Authors' Affiliations:
[1] Geriatric Unit, Department of Internal Medicine and Geriatrics, University of Palermo, Palermo, Italy;
[2] Faculty of Medicine and Surgery, Kore University of Enna, Enna, Italy.

Corresponding author:
Correspondence: Ligia J. Dominguez. Email: ligia.dominguez@unipa.it; ligia.dominguez@unikore.it

Funding source: For the preparation of this review the authors have received funding from the Fundación para la investigación biomédica de Córdoba (FIBICO) and FAES Farma, Bilbao Spain.

1. Background

The interest in vitamin D has increased in the last years since growing literature has reported that the deficit of vitamin D (i.e., hypovitaminosis D) could be associated with several negative health outcomes [1]. Moreover, hypovitaminosis D is a highly frequent condition, particularly in older populations [2].

The potential association between hypovitaminosis D and sarcopenia, i.e., the loss of muscle mass, poor physical performance, and low muscle strength has been reported by several epidemiological studies [3-5]. However, if the supplementation with vitamin D metabolites can prevent physical performance and muscle strength is still unclear.

Given this background, the present systematic review and meta-analysis aim to summarize the current state of the art of the effects of calcifediol on physical performance and muscle strength parameters.

2. Research question

The research question for this systematic review and meta-analysis is:

What are the benefits of calcifediol on physical performance and muscle strength parameters, based on current evidence derived from observational and intervention studies?

To guide the identification of adequate key words for building search strategies to search bibliographic databases, the research question was framed into PICOS (Participants, Intervention, Comparison, Outcome, Study type) format, knowing that there is no intervention in an umbrella review of observational studies. The research question formulated into PICOS format is as follows:

In people with hypovitaminosis D (P), compared baseline values (C), what is the effect of calcifediol (I) on muscle strength and physical performance parameters (O), based on evidence derived from intervention and observational studies(S)?

3. Methods

3.1 Protocol

We will also follow guidelines in the Cochrane handbook for systematic literature reviews to conduct the screening and selection of studies and the PRISMA statement [6].

3.2 Information sources and search strategies

For the conduct of this systematic review, several and relevant bibliographic databases will be comprehensively searched (with no date restriction), including Pubmed/Medline and Scopus, to which the University of Palermo has a full access.

We will build detailed and highly sensitive search strategies combining search terms (free vocabulary words and controlled vocabulary terms) from the main PICOS elements, tailored to the syntax of each of the other databases considered for this systematic review. All the detailed search strategies for all databases will be tested before engaging in the review process.

3.3 Eligibility Criteria

Inclusion criteria for this meta-analysis will be: (i) use of oral calcifediol; (ii) reporting information on physical performance (i.e., tests more depending on aerobic capacity than muscle power) [7] or muscle strength (i.e., tests more depending on muscle power than aerobic capacity) [7] outcomes; (iii) written in English. Studies will be excluded if: (i) did not include humans; (ii) lack of sufficient information for doing a meta-analysis. In this later case, the corresponding/first author of the article was contacted through e-mail. Study selection, at title/abstract and full-texts level, will be made using Rayyan (https://www.rayyan.ai/).

3.4 Study selection

We will follow recommendations in the Cochrane handbook for Systematic reviews to select studies that will be included in this review. The selections will be independently done by two review authors, with consensus meeting to discuss the studies for which divergent selection decision would be made by the two review authors. A third member of the review team will be involved, if necessary.

The studies selection process will involve, first, a selection based on title and/or abstracts, then a selection of studies retrieved from this first step based on the full-text manuscripts.

3.5 Data collection and data items

Items to be collected from the retrieved full-text articles are information for identification of the manuscript (e.g., first author name and affiliation, year of publication, journal name, title of the manuscript), data on the characteristics of the population considered, for individual observational studies (e.g., sample size, mean age, location, gender, etc.). For each article, we will extract also data on daily calcifediol supplementation (reported in microgram, ug), demographic information (mean age, females (%)), mean serum 25(OH)D levels reported for all studies in nmol/L at baseline and follow-up, follow-up in weeks.

3.6 *Data synthesis*

All analyses will be performed using STATA version 14.0 (StataCorp).

The primary analysis will compare the changes in physical performance and muscle strength parameters between follow-up versus baseline values. We then calculated the difference between the means of follow-up vs. baseline data using standardized mean differences (SMD) and their 95% confidence intervals (CIs). A random-effect model will be considered for all the analyses, being the less conservative and hypothesizing several clinical differences across the studies included [8]. We also plan, if possible, to compare the difference at follow-up between people supplemented with calcifediol and people taking placebo in muscle strength and physical performance parameters, having at least four studies available.

Heterogeneity across works will be assessed by the I^2 metric and χ^2 statistics. Given significant heterogeneity ($I^2 \geq 50\%$, $P<0.05$) a series of meta-regression analyses is planned, according to follow-up (weeks), mean age, percentage of females, serum 25(OH)D levels (baseline, follow-up, changes), dosages of calcifediol (10, 20, 30 ug/daily).

Publication bias will be assessed by visually inspecting funnel plots and using the Begg–Mazumdar Kendall tau[9] and the Egger bias test [10].

For all analyses, a P-value less than 0.05 will be considered statistically significant.

3.7 Ethical issues

This study will not involve patients or any human or animal material, and therefore does not imply any ethical issue.

References

1. Charoenngam, N.; Holick, M.F. Immunologic effects of vitamin D on human health and disease. *Nutrients* **2020**, *12*, 2097.

2. Dominguez, L.J.; Farruggia, M.; Veronese, N.; Barbagallo, M. Vitamin D sources, metabolism, and deficiency: available compounds and guidelines for its treatment. *Metabolites* **2021**, *11*, 255.

3. Annweiler, C.; Schott, A.-M.; Berrut, G.; Fantino, B.; Beauchet, O. Vitamin D-related changes in physical performance: a systematic review. *The journal of nutrition, health & aging* **2009**, *13*, 893-898.

4. Visser, M.; Deeg, D.J.; Lips, P. Low vitamin D and high parathyroid hormone levels as determinants of loss of muscle strength and muscle mass (sarcopenia): the Longitudinal Aging Study Amsterdam. *The Journal of Clinical Endocrinology & Metabolism* **2003**, *88*, 5766-5772.
5. Remelli, F.; Vitali, A.; Zurlo, A.; Volpato, S. Vitamin D deficiency and sarcopenia in older persons. *Nutrients* **2019**, *11*, 2861.
6. Liberati, A.; Altman, D.G.; Tetzlaff, J.; Mulrow, C.; Gøtzsche, P.C.; Ioannidis, J.P.; Clarke, M.; Devereaux, P.J.; Kleijnen, J.; Moher, D. The PRISMA statement for reporting systematic reviews and meta-analyses of studies that evaluate health care interventions: explanation and elaboration. *Journal of clinical epidemiology* **2009**, *62*, e1-e34.
7. Veronese, N.; Stubbs, B.; Trevisan, C.; Bolzetta, F.; De Rui, M.; Solmi, M.; Sarton, L.; Musacchio, E.; Zambon, S.; Perissinotto, E., et al. Poor Physical Performance Predicts Future Onset of Depression in Elderly People: Pro.V.A. Longitudinal Study. *Physical therapy* **2017**, doi:10.1093/ptj/pzx017.
8. DerSimonian, R.; Laird, N. Meta-analysis in clinical trials. *Controlled clinical trials* **1986**, *7*, 177-188.
9. Begg, C.B.; Mazumdar, M. Operating characteristics of a rank correlation test for publication bias. *Biometrics* **1994**, 1088-1101.
10. Egger, M.; Smith, G.D.; Schneider, M.; Minder, C. Bias in meta-analysis detected by a simple, graphical test. *Bmj* **1997**, *315*, 629-634.
